# Twenty-Month Regression Following Concurrent Conventional Whole-Brain Irradiation and Chemoimmunotherapy for ≥3.8 cm Cerebellar Metastasis From Small Cell Lung Cancer

**DOI:** 10.7759/cureus.43759

**Published:** 2023-08-19

**Authors:** Kazuhiro Ohtakara, Sosuke Arakawa, Makoto Nakao, Hideki Muramatsu, Kojiro Suzuki

**Affiliations:** 1 Department of Radiation Oncology, Kainan Hospital Aichi Prefectural Welfare Federation of Agricultural Cooperatives, Yatomi, JPN; 2 Department of Radiology, Aichi Medical University, Nagakute, JPN; 3 Department of Respiratory Medicine, Nagoya City University East Medical Center, Nagoya, JPN; 4 Department of Respiratory Medicine, Kainan Hospital Aichi Prefectural Welfare Federation of Agricultural Cooperatives, Yatomi, JPN

**Keywords:** multi-fraction, stereotactic radiosurgery, extensive stage, small cell lung cancer, pd-l1 inhibitor, consolidative thoracic radiotherapy, chemoimmunotherapy, whole-brain radiotherapy, large tumor, brain metastases

## Abstract

Standard whole-brain radiotherapy (WBRT) alone for large brain metastases (BMs) from small cell lung cancer (SCLC) has limited efficacy and durability, and stereotactic radiosurgery (SRS) alone for symptomatic posterior fossa BMs >3 cm with satellite lesions is challenging. Herein, we describe the case of a 73-year-old female presenting with treatment-naïve SCLC and 15 symptomatic multiple BMs, including a ≥3.8-cm cerebellar mass (≥17.7 cm^3^) and two adjacent lesions; otherwise, the SCLC was confined to the thorax.

The patient was initially treated concurrently with conventional WBRT (30 Gy in 10 fractions) without boost and chemoimmunotherapy (CIT) consisting of carboplatin, etoposide, and atezolizumab. Atezolizumab was excluded during irradiation. Five months after WBRT, the large cerebellar lesion had remarkably regressed, and the smaller lesions (≤17 mm) showed complete responses (CRs) without local progression at 20 months. However, six and 16 months after WBRT, the thoracic lesions had progressed, and although amrubicin was administered, four new BMs, including pons involvement, had developed, respectively. Despite the CRs of the four BMs following SRS (49.6 Gy in eight fractions) and the sustained regression of the thoracic lesions, meningeal dissemination and multiple new BMs were evident 3.5 months post-SRS. The small remnant of the large BM and/or newly developed BMs abutting the cerebrospinal fluid (CSF) space could have led to CSF dissemination, the presumed cause of the patient’s death.

Taken together, concurrent chemo-WBRT and subsequent CIT can provide excellent and durable tumor responses for SCLC BMs, but may not be fully sufficient for BMs ≥3.8 cm. Therefore, in cases with large lesions, focal dose escalation of the large lesions, consolidative thoracic radiotherapy, and dose de-escalation in the macroscopically unaffected brain region may prevent or attenuate CSF dissemination, new BM development, and adverse effects and thus should be considered.

## Introduction

Small cell lung cancer (SCLC) is an aggressive neuroendocrine malignancy with a high predisposition to developing brain metastasis (BM) [1,2]. The brain is a sanctuary site for metastatic cells owing to poor penetration of chemotherapeutic drugs across the blood-brain and blood-tumor barriers. Hence, external beam radiotherapy (EBRT) is an essential treatment option for BMs from SCLCs.

Compared to non-SCLC (NSCLC), whole-brain radiotherapy (WBRT) is preferred over stereotactic radiosurgery (SRS), especially for non-oligo BMs [3-5]. However, the efficacy and durability of standard WBRT (30 Gy in 10 fractions) are generally limited, particularly when the BMs are large [1, 3, 6]. Therefore, WBRT followed by a focal boost with SRS or three-dimensional conformal radiotherapy is considered in cases with large and/or oligo BMs [4,6,7]. Furthermore, WBRT has inherent detrimental effects on the brain and surrounding tissues in both the acute and late phases, especially in older patients [1,5,8,9].

Owing to the one-time application and the adverse effects of WBRT even with reduced prophylactic doses and driven by advances in systemic therapy, treatment strategies for small SCLC-BMs using upfront SRS with preservation of WBRT are attracting attention [5,8,9]. However, SRS for large (>3 cm) symptomatic posterior fossa BMs with satellite lesions inevitably increases dose spillage in the surrounding brain tissue and the risk of complications (e.g., obstructive hydrocephalus and tonsillar herniation due to radiation-induced edemata of the surrounding brain tissue and/or the tumor itself). Thus, the effective and safe performance of SRS in such scenarios remains challenging [10]. Furthermore, given the frequency and severity of microscopic brain invasion by SCLC-BMs [2,10,11], ensuring safe long-term local control of BMs >3.5 cm using only SRS in less than five fractions remains an unsolved conundrum [10].

The current standard first-line treatment for extensive-stage (ES)-SCLC is chemoimmunotherapy (CIT), consisting of etoposide-platinum (EP) chemotherapy combined with a programmed death ligand-1 inhibitor (atezolizumab or durvalumab) during and after chemotherapy [1,2,8,11]. In ES-SCLC cases with synchronous BMs (approximately 25% of cases) [2,11,12], CIT with EP plus durvalumab improves overall and progression-free survival more than chemotherapy with EP alone [2,11,12].

The response of BMs to CIT without EBRT remains unclear and is still under investigation [12]. For symptomatic and/or large SCLC-BMs, EBRT or other local therapy is generally performed before systemic therapy, whereas the efficacy and safety of concurrent WBRT and CIT are unresolved [1,2,8,13]. Lastly, for symptomatic large BMs not amenable to surgical removal, steroids must be administered to ameliorate and stabilize neurological symptoms before administration of immune checkpoint inhibitors (ICIs); however, steroids may suppress CD8+ cytotoxic T-cell activity and hamper the efficacy of the ICIs [14].

Herein, we describe the case of a 73-year-old female who presented with treatment-naïve ES-SCLC and multiple symptomatic BMs, including a >3.8-cm cerebellar lesion and two adjacent lesions. Concurrent conventional WBRT without local boost and CIT resulted in remarkable regression of the BMs without local progression for 20 months. The SCLC lesions in the thorax became refractory within six months after WBRT but did not progress further; however, the patient died, most likely due to cerebrospinal fluid (CSF) dissemination. We discuss possible measures for improving BM control, preventing CSF dissemination, and attenuating treatment-related neurotoxicity in the CIT era.

This report was part of the clinical study approved by the Clinical Research Review Board of Kainan Hospital Aichi Prefectural Welfare Federation of Agricultural Cooperatives (20220727-1).

## Case presentation

A 73-year-old female presented with unsteadiness, headaches, and difficulty walking. The patient was a current smoker, and the past medical history was unremarkable. No abnormalities were noted on screening performed two years earlier. The Karnofsky performance scale (KPS) score was 60 at consultation, and the patient was directly admitted to the hospital.

**Figure 1:**
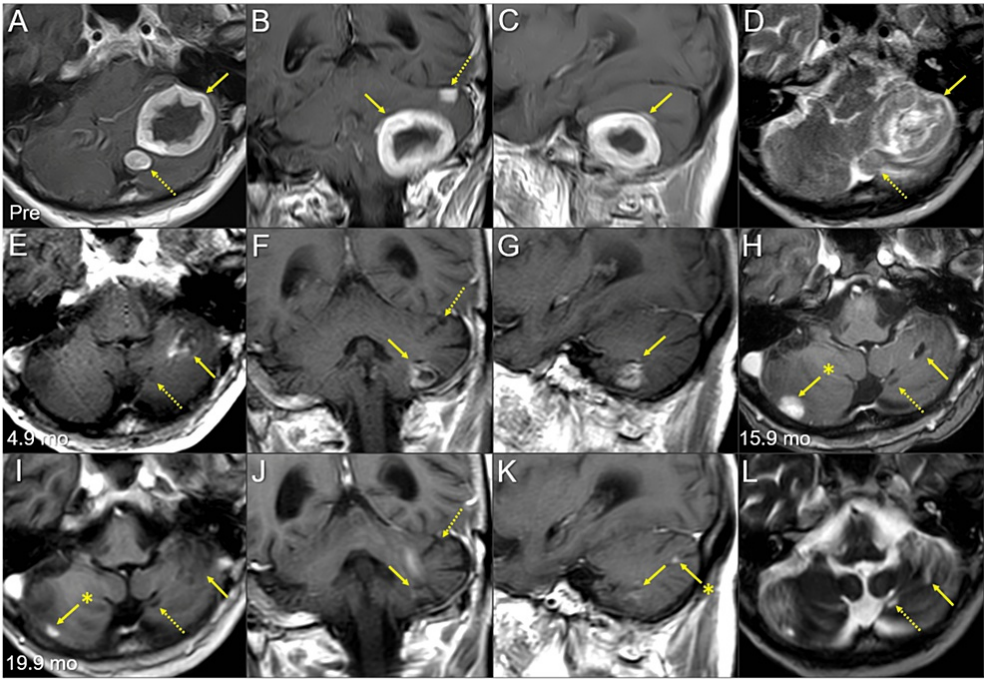
Magnetic resonance images of the posterior fossa lesions at the initial diagnosis and after whole-brain radiotherapy The images show contrast-enhanced (CE) T1-weighted images (WIs) (A-C, E-K); T2-WIs (D, L); axial images (A, D, E, H, I, L); coronal images (B, F, J); sagittal images (C, G, K); 13 days before (pre) the initiation of whole-brain radiotherapy (WBRT) (A-D); at 4.9 months (mo) after WBRT initiation (E-G); at 15.9 months (H); and at 19.9 months (I-L). (A-L) These images are shown at the same magnification and coordinates under co-registration and fusions. (A-D) A well-demarcated lesion with the dominance of the peripheral enhancement (3.8 cm in the maximum diameter, 17.7 cm^3^ in the volume) (arrows in A-C) on CE-T1-WIs is observed as the heterogeneous intensity mass (arrow in D) associated with mild perilesional edema on T2-WI. The lesion reaches the cerebellar surface at the ventrolateral and caudal sides and appears to extend beyond it in some areas. In the ipsilateral hemisphere, a 1.1 cm solid lesion (0.8 cm^3^) is located adjacent to the 3.8 cm lesion (dashed arrow in A, D), and a 0.9 cm lesion (0.3 cm^3^) is located less than 1 cm away from the 3.8 cm lesion (dashed arrow in B). (E-G) At 4.9 months, T2-WIs were unavailable. The large cerebellar lesion remarkably decreased in size, along with significant attenuation of the enhancing effect (arrows in E-G), and the two adjacent lesions (dashed arrows in E, F) regressed completely. (H) At 15.9 months, the large lesion was faintly enhanced at the periphery (arrow in H). The adjacent lesion is observed as the cavitary scar without enhancement (dashed arrow in H). A new enhancing lesion (arrow with an asterisk in H) appeared. (I-L) At 19.9 months, the enhancement of the large lesion was further attenuated (arrows in I-K), with the faint high-intensity scar (arrow in L) visible on T2-WI. None of the other two lesions showed local progression (dashed arrows in I, J, and L). The right cerebellar lesion regressed markedly (arrow with an asterisk in I). A new enhancing lesion (arrow with an asterisk in K) appeared.

**Figure 2:**
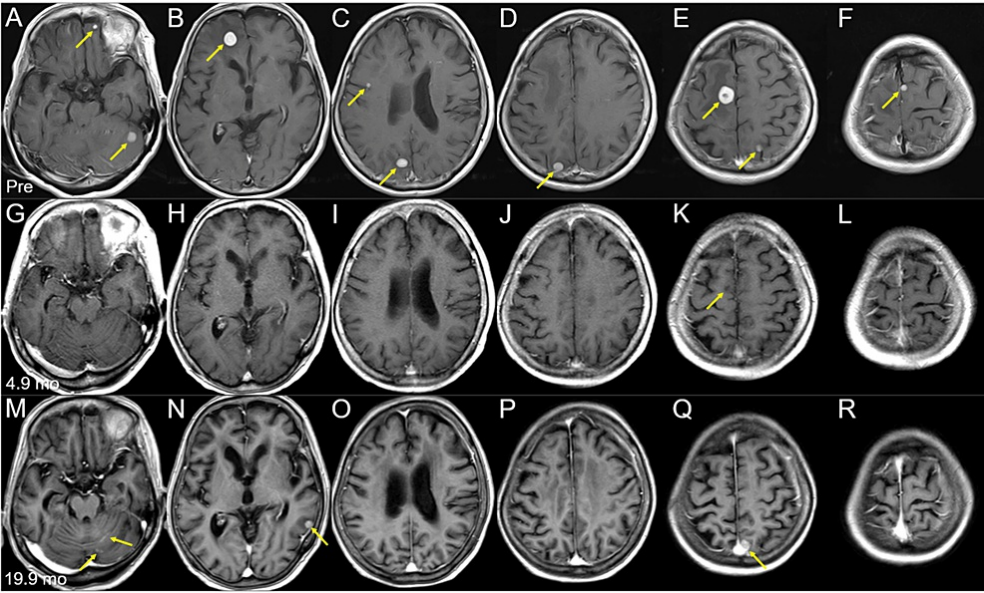
Brain magnetic resonance images of the other representative lesions at the initial diagnosis and after whole-brain radiotherapy The images show axial CE-T1-WIs (A-L); before (pre) the initiation of WBRT (A-F); at 4.9 months (mo) after WBRT initiation (G-L); and at 19.9 months (M-R). (A–R) These images are shown at the same magnification and coordinates under co-registration and fusions. (A-F) Nine out of the 15 lesions are visible as solid enhancing masses (arrows in A–F). (G-L) All nine lesions regressed completely, only leaving the cavitary scar in the right frontal lesion (arrow in K). (M-R) None of the nine lesions with complete responses showed local progression; however, multiple new lesions (arrows in N, Q) appeared, along with localized leptomeningeal dissemination (arrows in M). CE: contrast-enhanced; WIs: weighted images; WBRT: whole-brain radiotherapy

Contrast-enhanced (CE) magnetic resonance imaging (MRI) of the brain revealed 15 enhancing lesions, including a large cerebellar lesion (≥3.8 cm, 17.7 cm^3^), all of which were indicative of BMs (Figures 1, 2).

**Figure 3:**
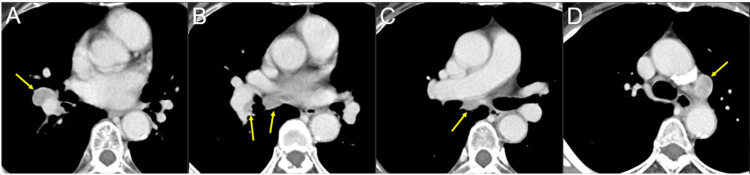
Chest computed tomography images at the initial diagnosis The images show axial CE computed tomography (CT) images (A-D). (A-D) The 2-cm well-demarcated mass lesion in the right middle-inferior perihilar region (arrow in A) and multiple lymph node swelling in the ipsilateral hilar and peribronchial (arrows in B), subcarinal (arrow in C), and subaortic (arrow in D) regions. CE: contrast-enhanced

Thoracic CE computed tomography revealed moderate centrilobular emphysema with a perihilar mass lesion and multiple swollen mediastinal lymph nodes (Figure 3).

**Figure 4:**
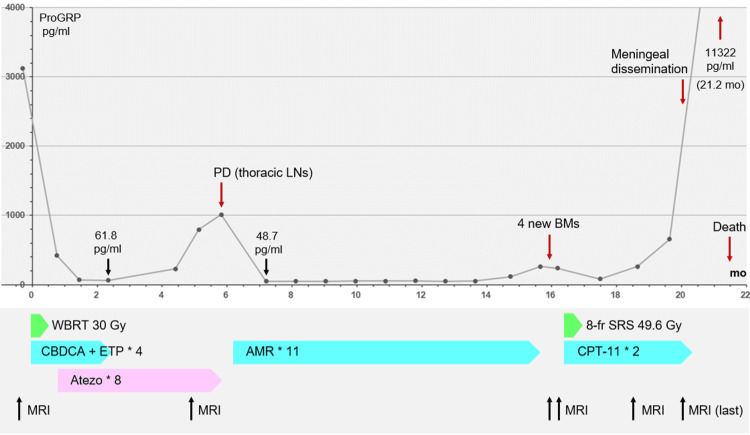
Summary of anti-cancer treatments after the initiation of whole-brain radiotherapy, along with the change in pro-gastrin-releasing peptide level and acquisition timing of brain magnetic resonance imaging CBDCA + ETP + atezolizumab: CBDCA (AUC 4 on day 1), ETP (80 mg/m^2^ on days one to three), and atezo (1200 mg/body on day 1); AMR monotherapy: AMR (35 mg/m^2^ on days one to three); CPT-11 monotherapy: CPT-11 (80 mg/m^2^ on days one, eight, and 15); day eight of the second course was the last administration. ProGRP: pro-gastrin-releasing peptide; mo: months; PD: progressive disease; LNs: lymph nodes; BMs: brain metastases; WBRT: whole-brain radiotherapy; 8-fr: eight-fraction; SRS: stereotactic radiosurgery; CBDCA: carboplatin; ETP: etoposide; AUC: area under the curve; atezo: atezolizumab; AMR: amrubicin; CPT-11: irinotecan; MRI: magnetic resonance imaging

The serum tumor marker elevations included pro-gastrin-releasing peptide (ProGRP) of 3121 pg/mL (normal limit, ≤80 pg/mL), neuron-specific enolase (NSE) of 19.4 ng/ml (≤15.0), and carcinoembryonic antigen of 11.3 ng/ml (≤4.1). The symptoms resolved after the administration of steroids and glycerol. Small cell lung cancer was pathologically diagnosed via a bronchoscopic biopsy. The clinical stage was IV B (cT1b N3 M1c) based on the tumor, node, and metastasis (TNM) classification according to the eighth edition of the Union for International Cancer Control (UICC). For the ES-SCLC, brain radiotherapy and CIT were concurrently performed [2,8,11,13]. The anti-cancer treatments are summarized in Figure 4; changes in ProGRP levels over time are also shown.

**Figure 5:**
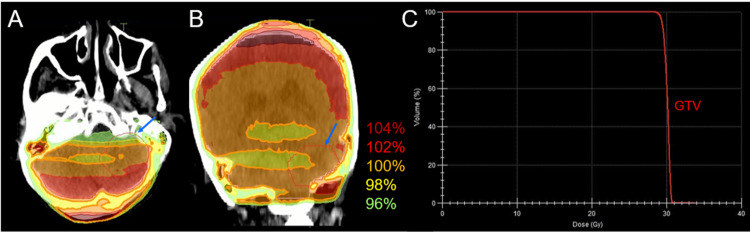
Dose distributions and dose-volume histogram of the large left cerebellar lesion in whole-brain radiotherapy The images show dose distributions (A, B); an axial image (A); a coronal image (B); and the dose-volume histogram (DVH) (C). (A, B) The representative % isodoses, normalized to 100% (30 Gy in 10 fractions) at the isocenter, and the gross tumor volume (GTV) contour of the left 3.8 cm cerebellar lesion (arrows in A, B). (C) The D_98%_, D_50%_, and D_2%_ of the GTV are 29.1 Gy (97.0%), 30.2 Gy (100.7%), and 30.7 Gy (102.3%), respectively, and 30 Gy encompasses 65% of the GTV. D_X%_: a minimum dose encompassing at least X% of the target volume (GTV).

Thirteen days after the initial MRI scan, the neurological symptoms had stabilized. At this time, WBRT (30 Gy in 10 fractions) with two-dimensional irradiation using two opposed fields was initiated; the prescription dose was assigned to the isocenter itself. The treatment platform was a multileaf collimator Agility® (Elekta AB, Stockholm, Sweden) mounted in a linac Infinity® (Elekta AB, Stockholm, Sweden) with a 6 megavoltage (MV) X-ray beam. The planning system was Monaco® (Elekta AB, Stockholm, Sweden), and the dose calculation algorithm was a collapsed cone with a calculation grid size of 2 mm. The treatment planning was performed by the predecessor. The dose distribution and planning parameters for the large cerebellar lesion are shown in Figure 5.

Etoposide plus carboplatin (EC) was administered one day after WBRT initiation, and atezolizumab was administered seven days after WBRT completion (Figure 4). These treatments were tolerated and completed by the patient without significant adverse events.

Following chemo-WBRT, the ProGRP level decreased to 61.8 pg/mL, and the thoracic lesions showed a partial response at the beginning of the fourth course of EC and the third course of atezolizumab. An MRI at 4.9 months after WBRT initiation revealed remarkable regression of the large cerebellar lesion, leaving only a small remnant, and complete responses (CRs) for the other 14 brain lesions (all ≤17 mm) (Figures 1, 2). However, during the eighth course of atezolizumab (5.8 months following WBRT), the disease was deemed refractory owing to the elevation of the ProGRP level and enlargement of the peribronchial lymph node metastases (Figure 4). Therefore, atezolizumab in the maintenance phase was replaced by amrubicin (AMR), which resulted in favorable tumor marker and imaging responses, with the nadir ProGRP response at 7.2 months (Figure 4). AMR was administered 11 times until progression.

**Figure 6:**
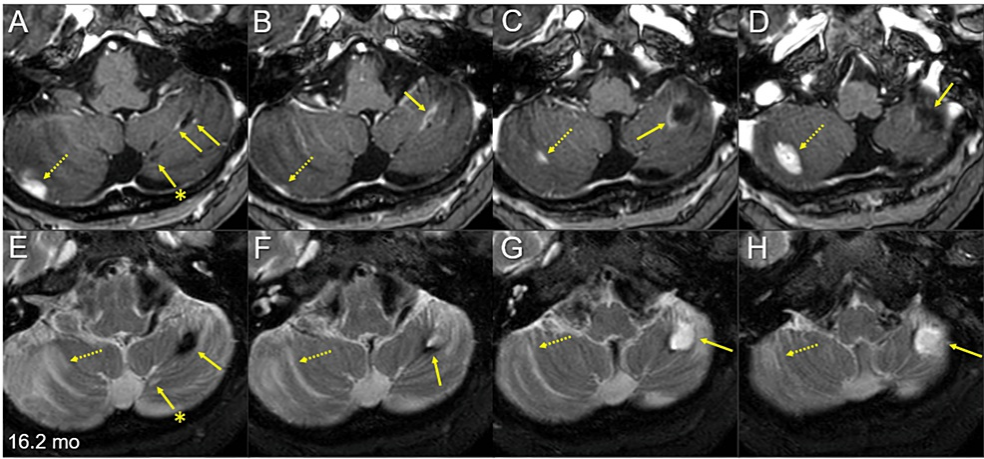
Multiple magnetic resonance images of the posterior fossa for the target definition of salvage stereotactic radiosurgery 16.2 months after whole-brain radiotherapy The images show axial CE-T1-WIs (A-D) and axial T2-WIs (E-H). (A-H) These images are shown at the same magnification and coordinates under co-registration and fusions. Alphabetically from cranial to caudal (A-D, E-H). The large left cerebellar lesion regressed remarkably, leaving the cavitary remnant (arrows in A-H), in which the periphery was partially enhanced (arrows in A-C) and partially hypointense on T2-WIs (arrows in E, F). The adjacent medial lesion regressed completely, only leaving the tiny cavitary scar (arrows with asterisks in A and E). Two discontinuous solid-enhancing lesions (dashed arrows in A-D) developed in the right cerebellar hemisphere, which is associated with perilesional edema (dashed arrows in E-H). mo: months; CE: contrast-enhanced; WIs: weighted images

**Figure 7:**
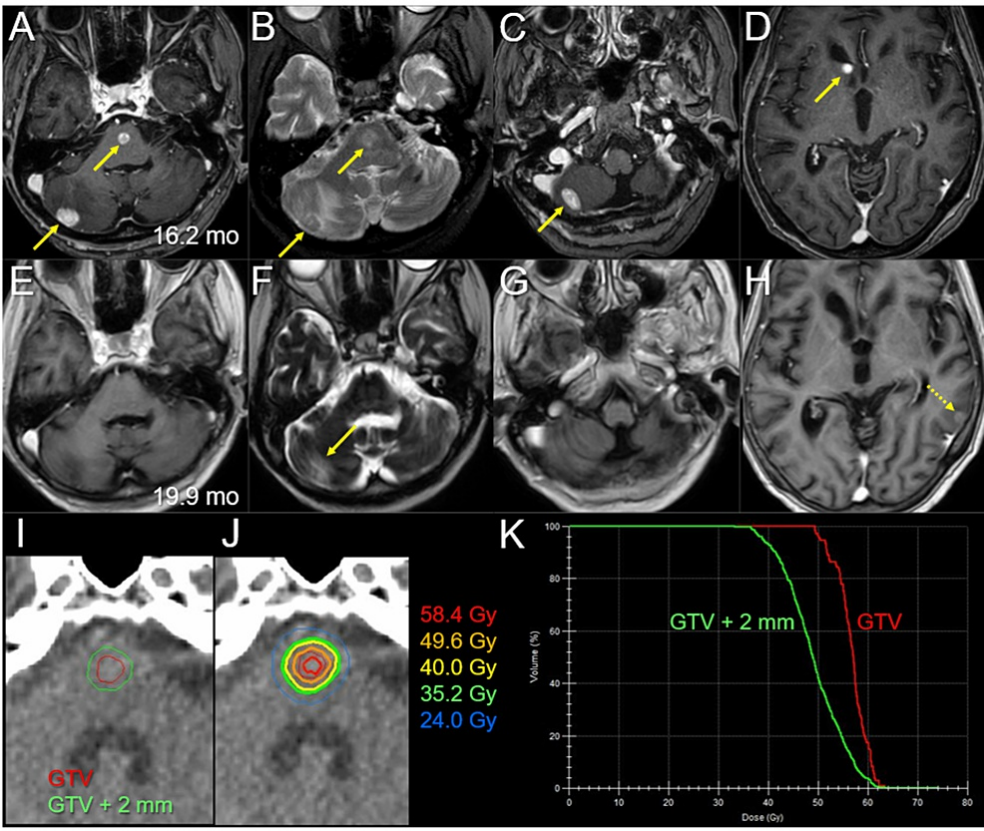
Magnetic resonance images before and after salvage stereotactic radiosurgery The images show axial CE-T1-WIs (A, C-E, G, H); axial T2-WIs (B, F); at 16.2 months (mo) after the initiation of WBRT (A-D); at 19.9 months (3.5 months after the initiation of SRS) (E-H); the target definition of the pons lesion (I); the dose distribution (J); the DVHs (K); and the pons lesion (I-K). (A–H) These images are shown at the same magnification and coordinates under co-registration and fusions. (A-D) Four solid-enhancing lesions (arrows in A, C, and D) are associated with perilesional edema (arrows in B). (E-H) The four lesions regressed completely, only leaving faint perilesional edema (arrow in F); however, a new lesion developed (dashed arrow in H). (J) Five representative isodose lines and the GTV contour are superimposed onto an axial CT image. CE: contrast-enhanced; WIs: weighted images; mo: months; WBRT: whole-brain radiotherapy; DVHs: dose-volume histograms; GTV: gross tumor volume; GTV + 2 mm: the reference volume generated by adding an isotropic 2-mm margin to a GTV

At 15.9 months, the ProGRP level increased without progression of the thoracic lesions. An MRI performed at this time revealed four new BMs, including the pons involvement and those contacting the CSF space, but no local progression of the initial 15 BMs (Figures 6-7).

Amrubicin monotherapy was switched to irinotecan (CPT-11) monotherapy at 16.4 months, and the four new BMs were treated via SRS (Figure 7); owing to brainstem involvement and prior WBRT, the prescription dose was 49.6 Gy in eight fractions to the gross tumor volume (GTV) margin with 67%-78% isodose coverage to ensure both efficacy and safety. Stereotactic radiosurgery was implemented with volumetric modulated arcs (VMA) using the Agility® on the Infinity®, in which the four lesions were simultaneously irradiated with a flattening filter-free mode of a 6 MV X-ray beam via a single isocenter [15]. The arc arrangement consists of one coplanar arc and two non-coplanar arcs with each arc length of 180º, which are allocated at 60º intervals to divide the cranial hemisphere evenly. The collimator angles for each arc are separately set to be 90º, 45º, and 135º. The dedicated software MIM Maestro^TM^ (MIM Software, Cleveland, OH, USA) was used for image co-registration, fusion, and contouring [10]. Monaco® was used to optimize the VMA plan (Table 1).

**Table 1:** Tumor characteristics and planning parameters for salvage eight-fraction stereotactic radiosurgery of the four new brain metastases *The dosimetric goals of treatment planning **The physical doses of 49.6 Gy and 35.2 Gy in eight fractions correspond to 80.4 Gy and 50.7 Gy, respectively, of the biological effective doses based on the linear-quadratic formula with an alpha/beta ratio of 10 (BED_10_). ***The %IDSs of 49.6 Gy relative to the D_max_ (100%). Rt: right; Lt: left; GTV: gross tumor volume; D_max_: maximum dose; BED_10_: the biological effective doses based on the linear-quadratic formula with an alpha/beta ratio of 10; D_98%_: a minimum dose encompassing at least 98% of the target volume; IDS: isodose surface; GTV + 2 mm: a reference volume generated by adding an isotropic 2-mm margin to a GTV

Location			Pons	Rt cerebellar (cranial)	Rt cerebellar (caudal)	Rt caudate head
GTV	Volume		0.11 cm^3^	0.75 cm^3^	0.53 cm^3^	0.16 cm^3^
	D_max_ (BED_10_)	≥65.9 Gy (120 Gy)*	63.5 Gy (113.9 Gy)	72.8 Gy (139.1 Gy)	74.1 Gy (142.7 Gy)	64.9 Gy (117.6 Gy)
	D_98%_ (BED_10_)	≥49.6 Gy (80 Gy)*	49.6 Gy (80.4 Gy)	51.0 Gy (83.5 Gy)	50.1 Gy (81.5 Gy)	52.0 Gy (85.8 Gy)
	49.6 Gy coverage**	≥98%***	97.7%	99.2%	98.9%	100%
	%IDS***	≤75%***	78.1%	68.1%	66.9%	76.4%
GTV + 2 mm	D_98%_ (BED_10_)	≥35.2 Gy (50 Gy)*	37.4 Gy (54.9 Gy)	38.1 Gy (56.3 Gy)	36.6 Gy (53.3 Gy)	40.6 Gy (61.2 Gy)
	35.2 Gy coverage**	≥95%***	99.8%	99.9%	99.2%	100%

**Figure 8:**
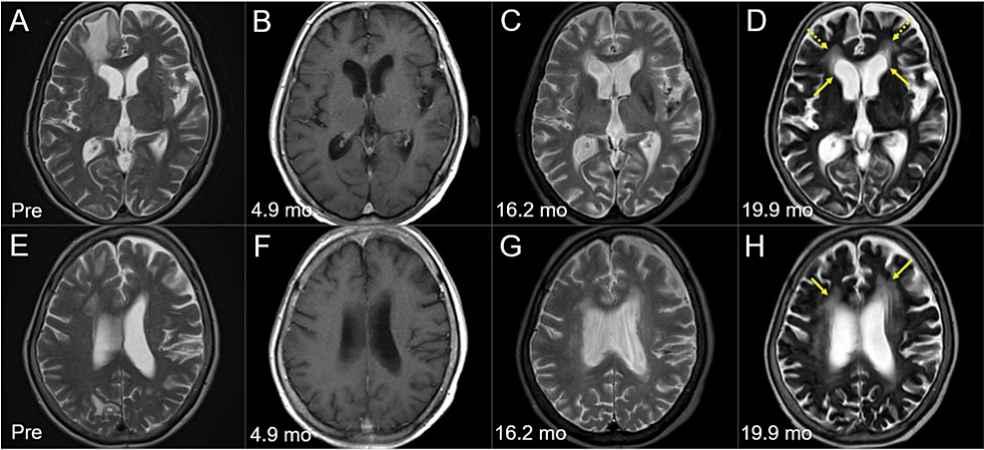
Morphological changes of the brain on magnetic resonance images before and after whole-brain radiotherapy The images show axial T2-WIs (A, C-E, G, H); axial CE-T1-WIs (B, F); before WBRT (Pre) (A, E); at 4.9 months (mo) after the initiation of WBRT (B, F); at 16.2 months (C, G); and at 19.9 months (D, H). (A-H) These images are shown at the same magnification and coordinates under co-registration and fusions. (B, F) T2-WIs were unavailable at 4.9 months. (C, D, G, H) Dilatation of the lateral ventricles (arrows in D) with high-intensity change in the periventricular deep white matter (dashed arrows in D, arrows in H) and widening of the cortical sulci and the Sylvian fissures reflect the progression of brain atrophy and the degenerative changes of the parenchyma. WIs: weighted images; CE: contrast-enhanced; WBRT: whole-brain radiotherapy

The patient was treated for febrile neutropenia 19 days after the initiation of CPT-11 monotherapy and SRS. An MRI at 2.1 months after CPT-11/SRS initiation showed CRs for the four new lesions, with sustained regression at 3.5 months (Figure 7). However, an MRI at 20 months after WBRT (3.5 months after SRS) revealed limited meningeal dissemination along with multiple new lesions (Figure 2), although none of the initial 15 BMs had progressed locally (Figures 1, 2, 6). Cross-temporal comparison of the brain morphology on serial MRI scans revealed obvious progression of ventricular dilatation and widening of the cortical sulci, both of which were mainly attributed to a decrease in brain volume (Figure 8).

Owing to the presence of CSF dissemination, resistance to CPT-11 monotherapy, and declining performance status, the patient was transitioned to palliative care. One month later, oral intake became difficult, and the ProGRP level increased precipitously (NSE at 21.2 months, 38.0 ng/mL), and the patient died 21.5 months after the initiation of concurrent chemo-WBRT (Figure 4).

## Discussion

The present case describes the challenging treatment of at least 15 synchronous SCLC-BMs, including a symptomatic ≥3.8-cm cerebellar lesion, in a current smoker in her early 70s. The cerebellar lesion was likely >4 cm (>20 cm^3^) at the start of WBRT given the 13-day interval between its initial detection and WBRT initiation, although the extracranial tumor burden was asymptomatic and confined to the thorax [3,11,16].

As proposed in 2018, the brain metastases from SCLC (BMS) score serves as the prognostic score for WBRT-treated SCLC-BMs [17]. The BMS score in the present case was "group II" (two points), and, assuming the extracranial lesion was stable, the estimated median survival time was 6.6 months. According to the National Cancer Database Survey from 2010 to 2014, the median survival times for older patients (≥75 years) with SCLC-BMs who received WBRT alone, chemotherapy with WBRT, and chemotherapy without WBRT were 1.9, 5.6, and 6.4 months, respectively; the difference between WBRT with/without chemotherapy was not significant [16]. Thus, in the pre-CIT era, the outcomes of older patients with SCLC-BMs, especially those with low KPS scores, were usually dismal, irrespective of treatment selection.

Against this background, the excellent and durable responses of the initial BMs in the present case likely reflect the significant enhancement of conventional WBRT by concurrent EC treatment and subsequent EC treatment plus an ICI. Achieving similar efficacy and safety when using SRS (less than five fractions) for large BMs is not as straightforward. Among the 36 pre-CIT era cases of ES-SCLC reported by Chen et al., in which the brain was the sole distant metastatic site, WBRT (30 Gy in 10 fractions) with concurrent etoposide and cisplatin achieved a CR in less than half (44%) [18].

Although early and sufficient administration of steroids was required before administration of atezolizumab in the present case, Sorial et al. found no significant association between corticosteroid use before CIT and worse outcomes in patients with ES-SCLC or non-SCLC [14]. However, determining the efficacy of atezolizumab was difficult owing to the gap of 4.9 months between WBRT initiation and MRI re-evaluation; this is a significant limitation of this report.

The efficacy and safety of concurrent WBRT and CIT remain unclear. In the present case, CRs were sustained for 20 months for all BMs ≤17 mm; this indicates that microscopic BMs present at the initiation of WBRT can be completely eradicated by concurrent reduced-dose WBRT (<30 Gy) and CIT, which may attenuate WBRT-induced neurocognitive decline. In our case, chemo-WBRT followed by CIT resulted in remarkable regression of the large (>3.8 cm) BM but did not produce a CR. Residual viable tumors can eventually regrow and/or disseminate unless controlled by systemic therapy or additional EBRT. Therefore, focal dose escalation in the initial EBRT application likely improves local control, preferably achieving a CR, and a modest dose increase of ≥10%-20% may be sufficient. Integration of VMA with a simultaneous-integrated boost into WBRT enables focal dose escalation and dose reduction in affected and unaffected brain regions, respectively [1,8].

In the present case, no obvious progression of the thoracic lesions was evident until just before death; hence, death was attributed to CSF dissemination. Prevention of CSF dissemination is essential for improving the outcomes of patients with SCLC-BMs. Potential sources of CSF dissemination in the present case include the small tumor remnant fronting the regressed cavity of the left cerebellar lesion and the development of new BMs in the right caudate head and cerebellar surface, all of which contact the CSF space. Focal dose escalation in cases with large lesions would help eradicate one of the potential sources of dissemination. In cases with metachronous BMs, early detection and appropriate execution of salvage SRS is required before tumor seeding occurs.

To prevent or attenuate the development of new metachronous BMs, improving extracranial disease control is important. Consolidative thoracic radiotherapy for a partial response after CIT remains controversial [1,2,13,19]; however, some studies reported positive survival outcomes in patients who received this serial treatment [19]. Consolidative thoracic radiotherapy with a modest dose (e.g., 30 Gy) and image-guided VMA was a potential treatment option for the regressed thoracic lesions during the maintenance phase with atezolizumab in the present case.

Salvage re-irradiation of brainstem metastases developing after prior WBRT (30 Gy) is also challenging. The prescribed dose for salvage re-irradiation is commonly and frequently low owing to the risk of radiation-induced brainstem injury [15,20]. However, insufficient local control or tumor regrowth following SRS for brainstem metastases inevitably leads to significant impairment of neurological function, for which efficacious and safe salvage treatment options are extremely limited [15]. In the present case, sufficient GTV coverage with a BED_10_ of 80 Gy in eight fractions and concurrent CPT-11 administration achieved CRs without ARE [15] for all treated lesions. However, due to the short observation period of 3.7 months, we could not conclude the validity of the treatment scheme.

Nevertheless, optimal strategies combining EBRT with systemic therapy for synchronous or metachronous SCLC-BMs in the CIT era should be reconsidered to further improve anti-tumor efficacy while preserving neurocognitive function [5,11,13].

## Conclusions

Concurrent conventional WBRT without boost and EP administration followed by CIT can yield excellent and durable tumor responses in patients with SCLC-BMs, even those with BMs ≥3.8 cm. Using this regimen, a CR can be maintained for 20 months if the BM is ≤17 mm; however, it may be insufficient for BMs ≥3.8 cm. In cases with large lesions, modest focal dose escalation should be considered to improve BM control and prevent CSF dissemination, along with dose de-escalation in the unaffected brain region to attenuate the decline of neurocognitive function. To prevent the development of new metachronous BMs, consolidative thoracic radiotherapy may be a potential treatment option for responders with active disease confined to the thorax following CIT.
